# John Devlin: Nova Cantabrigiensis

**DOI:** 10.1017/S2045796019000878

**Published:** 2020-01-27

**Authors:** Linda Rainaldi

**Affiliations:** Independent Researcher, Vancouver, Canada

**Keywords:** Art brut, contemporary art, intuitive art, outsider art

John Devlin (b. 1954) has spent a significant part of his life seeking to articulate the mystery of a sublime moment he experienced at Cambridge University. After exploring architecture and the world of finance in Nova Scotia, Canada, he decided he was best suited for the priesthood and departed for Cambridge to study theology in 1979. Due to a breakdown in his health, he left his studies and returned home the following year. That's when he started drawing. Devlin's admittedly unscientific belief is that the drug Tegretol released his undiscovered artistic abilities. Using his younger sister's crayons, coloured pencils and ball point pens, he began his Nova Cantabrigiensis project and the stacks of drawings started piling up. In hindsight, there was a reason he fell ill at Cambridge – it was to find his true vocation. He didn't plan to be an artist; it just happened.

**Fig. 1.**
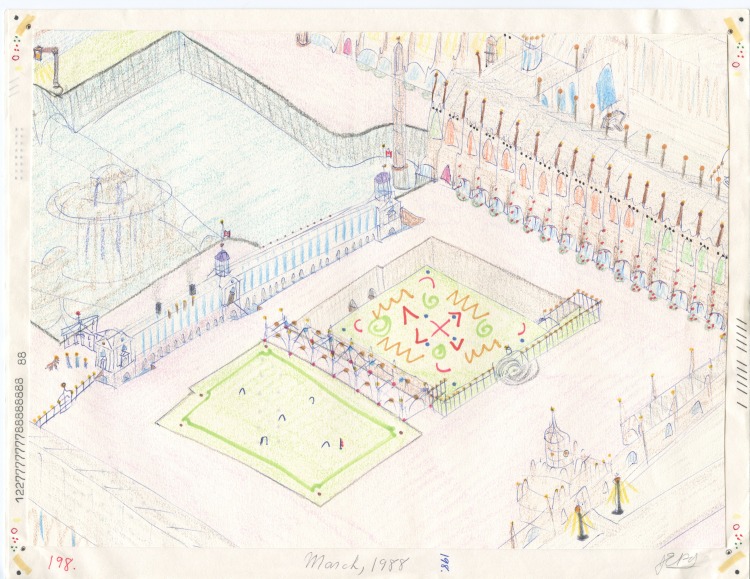
John Devlin, *Study of King's College, Cambridge*, 1988, Mixed media on paper, 21.6 × 28 cm. Collection Henry Boxer Gallery.*

Nova Cantabrigiensis is a utopian vision of a university, modelled on Cambridge, but located in the Minas Basin in Nova Scotia, a special area of the Bay of Fundy, home to the most dramatic tidal range in the world.

The campus was a fictitious world that Devlin would like to inhabit, and he drew himself into many of his pictures. Like a child absorbed in a fantasy world of his own creation, he was *in* Cambridge again, walking past grand buildings with a dog at his side. Nova Cantabrigiensis, however, would be a reformed institution, free from the inflated egos of the old-school culture as well as the debilitating effects of study and examinations. And it would also be a retirement community of sorts, where one could stay after graduation. Devlin worked fervently on this project from 1984 to 1988, drawing his own version of Cambridge's illustrious buildings, making them more monumental. His art, he says, is about structure and the endless search for beauty.

King's Chapel, in particular, is the subject of countless drawings because it's where Devlin experienced an acute aesthetic response, a feeling he still struggles to understand. He remembers what it felt like the first time he walked into the courtyard, entered the chapel and heard the choir perform. He has since returned to visit King's Chapel, but could not recapture that moment. He continues to draw the building, hoping to rediscover its secret power. He would like to isolate the essence of that first experience so as to relive it over and over again.

**Fig. 2.**
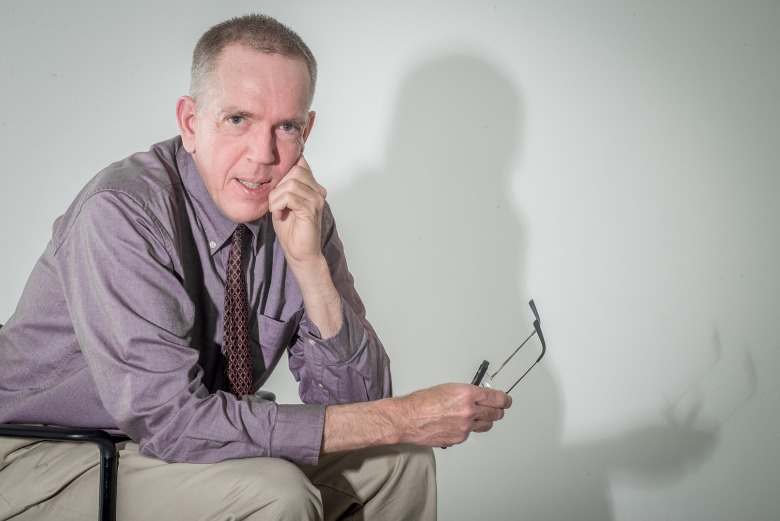
John Devlin, Photo by Ken Kam.

Devlin recalls his first encounter with mental illness – a baffling world where normal laws did not apply. It was an enlightening, but terrifying experience. He has yet to understand the laws that governed his psychotic universe. ‘I had wisdom, but spoke in riddles. People were mysteries to me, and I found language no longer communicated what I wished. I was forced to find other ways of communication, and I turned to sketches and diagrams to describe my inner state’ (Devlin, 2016). As many others have described, the elements of speech have no meaning and connect to nothing; that leads to a profound sense of disorder. Words become a bewildering collection of sounds, with no code to decipher them (Rogers, 2016).

Devlin continues to explore the ideal of perfect architecture and admits to still being obsessed with numbers. He employs reductive notations in his drawings, a shorthand script for information like the length of a wall, the number of doorways, and so on. He also abstracts algorithms from the completed drawings, as well as subliminal forms, hidden codes and equations, but hides all these notes on the back of the page, leaving the image to speak for itself. Devlin has put aside the Nova Cantabrigiensis project, but continues to explore the structure of King's Chapel, most recently in the form of an annulus. The east side bends back on itself to join the west, creating a circular space.

Devlin has amassed a vast collection of over 1800 works, some of which are featured in a publication of his Nova Cantabrigiensis project (Robinson, 2011). Although he still draws regularly – sometimes with a sense of urgency and at other times laboriously – Devlin is now exploring new mediums. He is currently working with gold and silver leaf, inspired by the dazzling splendor of Klimt's *Portrait of Adele Bloch-Bauer I*. Reminiscent of Byzantine gold mosaics, bathing the universe in divine light, Devlin touches the sacred with his careful application of gold squares to paper. He describes a life of solitude, finding joy in creating art in his room. Nothing can beat that experience, he says. Devlin had never heard of outsider art until his work was exhibited at the Technical University of Nova Scotia school of architecture in 1988. He is puzzled by the label and would prefer that his work not be framed in an academic light. He is just an artist, born to toil over his work, striving to leave the world a more beautiful place.

## About the Author

Linda Rainaldi writes on the subjects of both law and outsider art. Her most recent art publication was *The Stuff of Life – The Sculptures of Jordan MacLachlan*, in Raw Vision #94, Summer 2017. She is writing a book about Canadian outsider art, to be published in 2020. She lives in Vancouver, Canada.

Carole Tansella, *Section Editor*
