# John Devlin: Nova Cantabrigiensis – ADDENDUM

**DOI:** 10.1017/S2045796020000220

**Published:** 2020-02-27

**Authors:** Linda Rainaldi

**Affiliations:** Independent Researcher, Vancouver, Canada

In the aforementioned paper, figure captions were omitted from the text and the appropriate owners were not credited. The figures should read as follows:

**Fig. 1.** John Devlin, *Study of King's College, Cambridge*, 1988, Mixed media on paper, 21.6 × 28 cm. Collection Henry Boxer Gallery.

**Fig. 2.** John Devlin, Photo by Ken Kam.

The affiliation of the author is also modified to ‘Independent Researcher, Vancouver, Canada’. The author and publisher apologise for these omissions.
